# Theoretical investigation of hybrid nanomaterials transient flow through variable feature of Darcy–Forchheimer space with exponential heat source and slip condition

**DOI:** 10.1038/s41598-022-17988-1

**Published:** 2022-09-05

**Authors:** Ikram Ullah, Yahya Alajlani, Amjad Ali Pasha, Mohammad Adil, Wajaree Weera

**Affiliations:** 1 Department of Sciences and Humanities, National University of Computer and Emerging Sciences, Peshawar, 25000 KP Pakistan; 2 Department of Physics, Faculty of Science, Jazan University, Jazan, Saudi Arabia; 3 Aerospace Engineering Department, King Abdulaziz University, Jeddah 21589, Saudi Arabia; 4 Mechanical Engineering Program, Physical Science and Engineering Division, King Abdullah University of Science and Technology, Thuwal, 23955-6900 Saudi Arabia; 5 KAUST Clean Combustion Research Center, King Abdullah University of Science and Technology, Thuwal, 23955-6900 Saudi Arabia; 6 Department of Mathematics, Faculty of Science, Khon Kaen University, Khon Kaen, 40002 Thailand

**Keywords:** Nanoscale materials, Fluid dynamics, Renewable energy, Applied mathematics

## Abstract

Nanomaterials have achieved remarkable importance in cooling small electronic gadgets like akin and microchips devices. The role of nanoparticles is essential in various aspects, especially in biomedical engineering. Thus hybrid nanomaterials is introduced to strengthen the heat exchangers' performance. In view of the above practical and existing applications of nanomaterials. Our aim is to examine the consequences of Darcy–Forchheimer's radiative and Hall current flow of nanomaterials over a rotating porous disk with variable characteristics. Stretching disk accounting for the slip condition. Nanoparticles ZnO and CoF_2_O_4_ are dispersed in based fluid water. The present model is utilized for thermo-physical attributes of hybrid nanomaterials with the impact of shape factor. Transformations convert the modeled PDEs into ODEs. The obtained highly non-linear system is tackled numerically by the NDSolve technique through the software Mathematica. The outcomes of significant variables against different profiles are executed and elaborated in detail. Obtained results show that both nano and hybrid nanofluid radial velocity have reverse behavior against variable porosity and permeability parameters, whereas it decays for larger Forchheimer numbers. Further, it is worthy to point out that, hybrid nanophase has a higher impact on distinct profiles when compared with nano and common liquid phases.

## Introduction

An advancement in heat transportation phenomenon through liquids is the main issue in various industrial and technologically system. Because such liquids are widely utilized in many industries. These liquids are addressed to be functioning liquid in machinery system, electronic devices and have several significant applications like thermal energy accumulation and elimination from one section of machine to another. However the low thermal features of these fluids is the key problem facing in the transportation of heat phenomenon. In order to resolve this complexity, various scientist added some solid particles having size less than 100 nm in the traditional liquids which shows high thermal characteristics when compared with base liquid. This special material is term as nano-liquids. Different researchers utilized different nanoparticles in the common liquid to explore the thermal features of liquid with various aspects. Hybrid nano materials are basically the composition of more than one nano-particles in base liquid. This nano-liquid gives highly efficient energy compared to that of common nano-liquid. In this regard Ramesh et al.^1^ developed the mathematical relations describing the hybrid nanomaterials flow. Theoretical analysis of both nano and hybrid nano fluid was scrutinized by kumar et al.^2^. Few novel attempts readings hybrid nanomaterials are^3–30^.

Hall phenomena have gained remarkable attention owing to their uses in astrophysical, geophysical and engineering problems like Hall aspect in sensors and Hall accelerators etc. In the existence of strong magnetic field or in rarefied medium, the features of Hall current cannot be ignored. The trend of current for the use of MHD is towards strong field of magnet (In this case the effect of electromagnetic for is remarkable) and trend to less density of field like in nuclear fusion space light. In view of above constrain, the Hall current becomes significant^30^. The important use of Hall current in medical science i.e. in MRI, ECG etc. Katagiri^31^ analyzed the impact of Hall current in MHD flow over a semi-infinite plate. Here applications of Hall current is analyzed. The results of the analysis revealed that Hall parameter has decreasing effect on blood flow, but opposite effect is noted with the increasing Hartmann number^32^. Mahdy et al.^33^. discussed the features of Hall current on micro-temperature in a semi-conductor space. It has been examined from the study that variation in Hall current have a significant impact on velocity. Sabu et al.^34^ statistically explored the Hall current phenomenon on ferro-liquid flow through inclined channel. Hall current has a decay effect on skin friction. The 3D Casson magnetized nanomaterilas flow with ion slip and Hall features is examined by Ibrahim and Anbessa^35^. Currently Ullah et al.^36^ discussed the ion slip and Lorentz force effects on peristaltic channel.

Heat transfer improvement in hybrid nanomaterials flow through porous space have numerous utilization in the various areas like petroleum, environmental, civil and biomedical engineerings and agricultural etc. Production of oil and gas from reservoirs, water pollution through toxic liquids, irrigation and drainage, infrastructure construction, bio sensors and petrochemicals are the some significant applications. Flow through porous systems has been modeled by utilizing Darcy’s law extensively. The flow converted to non-Darcian, when the Reynolds number exceeds unity, because the inertial aspect cause extra hydrodynamic head loss. In 1901 a Dutch researcher Forchheimer, dispense his ideas and expressions more extensively. Additionally he admitted the squared velocity term in the momentum expression to compute the inertial forces^37^. Muskat^38^ named this term as Forchheimer term. Few development in this area can be viewed^39–44^. Heat transportation analysis in variable porous space is discussed by Vafai^45^. After that Vafai et al.^46^ performed the experimental inspection of flow via porous space subjected variable features. Ress and Pop^47^ explored the variable permeability effects on a vertical free surface. Hayat et al.^48^ examined the transient nanomaterial flow through porous regime with variable characteristics. Very recently, Ullah et al.^49^ the nanomaterials flow through Darcy–Forchheimer (DF) space with varying permeability.

This work presents the Hall current and Lorentz force on flow of hybrid nanofluid ($$CoF_{2} O_{4} - ZnO/water)$$ over slippery and rotating porous disk with variable permeability. The key motivations of executing presents study are summarized as:

- Explore the Lorentz force and Hall current applications for hybrid nanoliquid flow subject to a porous disk.
- Novel features of $CoF_{2} O_{4}$ and $ZnO$ conveying water hybrid nanomaterials.
- Darcy–Forchheimer law with variable porosity and permeability features is considered as a novelty.
- To investigate the thermal performance of hybrid nanomaterial with EHS, dissipation and radiation impacts.
- To discuss the rate of heat transportation with different shape of nanoparticles.
- Slippery constrains are imposed to examine the fluid flow.
- The numerical simulations are executed by utilizing the built in shooting techniques.
- The inspection of liquid with addition of two different nanoparticles is useful in machinery system, electronic devices, medical equipment’s and treatment of diseases.

## Problem formulation

**Figure 1:**
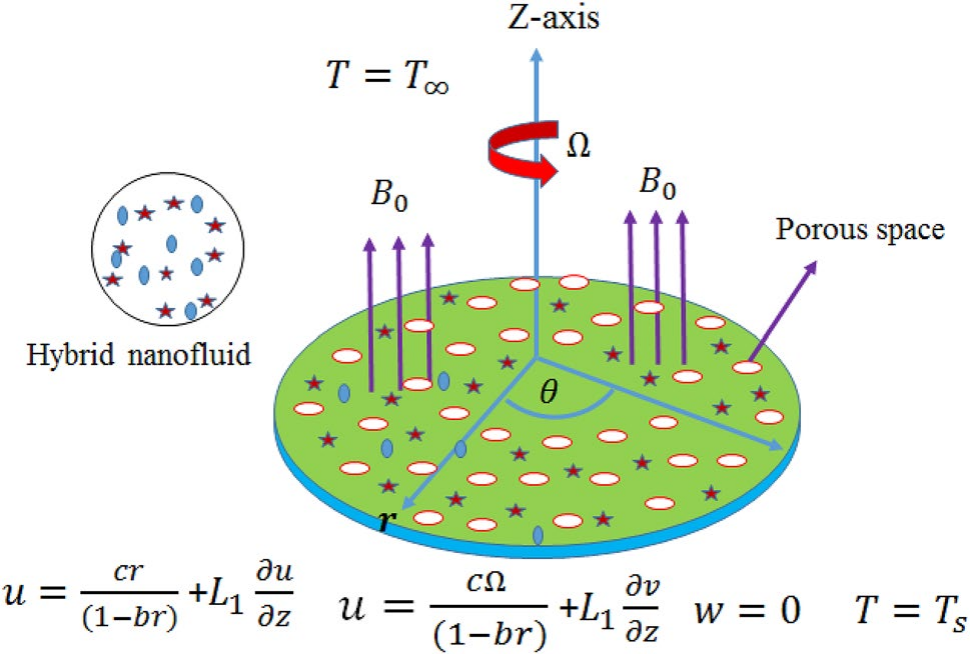
Flow physical configuration.

Consider the magnetized hybrid nanofluid flow through a spinning and slippery porous disk. Fluid flow via Darcy–Forchheimer porous space is assumed with variable features. The porous disk rotates with an angular velocity ($\Omega$) and stretch at $z = 0$ (see Fig. 1), which causes the hybrid nanomaterials motion. The surface of porous disk is sustain at temperature $T_{s}$ and ambient temperature is $T_{\infty }$. The hybrid nanomaterials is the suspension of two kinds of nanomaterials $CoF_{2} O_{4}$ and $ZnO$ in water. Hall current is the result of higher magnetic field applied normally to the disk. Impact of radiation, EHS and dissipation are additionally considered to examine the variation in temperature gradient comprehensively. Keeping in mind the aforementioned assumptions, the Hall current and Ohm’s law relations are:

1 $$B\left( t \right) = \frac{{B_{o} }}{{\left( {1 - bt} \right)^{{\tfrac{1}{2}}} }},u = \frac{cr}{{\left( {1 - bt} \right)}}+\, L_{1} \frac{\partial u}{{\partial z}},v = \frac{r\Omega }{{\left( {1 - bt} \right)}}+\, L_{1} \frac{\partial v}{{\partial z}},T_{s} = T_{o} - T_{ref} \frac{{r^{2} \Omega }}{{v_{f} \left( {1 - bt} \right)^{{\tfrac{3}{2}}} }}.$$

2 $$\frac{{\omega_{e} \tau_{e} }}{{B_{o} }}\left( {J \times B} \right) + J = \,\sigma \left[ {\frac{1}{{en_{e} }}\nabla p_{e} + \mu_{e} (V \times B)} \right],\,$$

The electric field is considered to be zero, taking the weakly ionized gas with negligible slip conditions and thermoelectric pressure. So the Hall current in component form are:

3 $$J_{r} = \frac{{\mu_{e} \sigma B_{o} }}{{\left( {1 + m^{2} } \right)}}\left( {mv - u} \right),\,\,$$

4 $$J_{\varphi } = \frac{{\mu_{e} \sigma B_{o} }}{{\left( {1 + m^{2} } \right)}}\left( {mv + v} \right),\,$$

In above equations, $B$ manifests the magnetic induction, $J$ the vector for current density, $(T_{s} ,\,\,T_{ref} ,\,\,T_{o} )$ the surface, constant reference, origin temperatures respectively, ($b,\,\,c,\,$) denote stretching rates, $B_{o}$ magnetic field strength,$\,\omega_{e}$ cyclotron frequency of electron, $\tau_{e}$ electron collision time, $p_{e}$ the electronic pressure, $\mu_{e}$ the magnetic permeability, $n_{e}$ the number of density of electron, $\sigma$ electrical conductivity of fluid and $m$ the Hall current parameter. Hall current and the electrical conductivity and hall current expressions are $\sigma = \frac{{e^{2} n_{e} t_{e} }}{{m_{e} }}$ and $m = \omega_{e} \tau_{e}$.

The flow expressions for current analysis are^30,49^:

5 $$\frac{\partial w}{{\partial z}} + \frac{1}{r}\frac{\partial }{\partial r}\left( {ur} \right) = 0,\,$$

6 $$\frac{\partial u}{{\partial t}} - \frac{{v^{2} }}{r} + w\frac{\partial u}{{\partial z}} + u\frac{\partial u}{{\partial r}} = v_{hnf} \left( {\frac{{\partial^{2} u}}{{\partial z^{2} }}} \right) - \frac{{\sigma_{hnf} B_{o}^{2} }}{{\rho_{hnf} \left( {1 + m^{2} } \right)\left( {1 - bt} \right)^{\frac{1}{2}} }}\left( {u - mv} \right) - \frac{{\varepsilon (z)\nu_{hnf} }}{{k^{**} (z)}}u - \frac{{\varepsilon^{2} (z)C_{b} }}{{\sqrt {k^{**} (z)} }}u\sqrt {u^{2} + v^{2} } ,$$

7 $$\frac{\partial v}{{\partial t}} + u\frac{\partial v}{{\partial r}} + \frac{uv}{r} + w\frac{\partial v}{{\partial z}} = v_{hnf} \left( {\frac{{\partial^{2} v}}{{\partial z^{2} }}} \right) - \frac{{\sigma_{hnf} B_{o}^{2} }}{{\rho_{hnf} \left( {1 + m^{2} } \right)\left( {1 - bt} \right)^{\frac{1}{2}} }}\left( {v + mu} \right) - \frac{{\varepsilon (z)\nu_{hnf} }}{{k^{**} (z)}}v - \frac{{\varepsilon^{2} (z)C_{b} }}{{\sqrt {k^{**} (z)} }}v\sqrt {u^{2} + v^{2} } ,$$

8 $$\frac{\partial T}{{\partial t}} + u\frac{\partial T}{{\partial r}} + w\frac{\partial T}{{\partial z}} = \frac{{k_{hnf} }}{{(\rho c_{p} )_{hnf} }}\left( {\frac{{\partial^{2} T}}{{\partial z^{2} }}} \right) - \frac{1}{{(\rho c_{p} )_{hnf} }}\frac{{\partial q{}_{r}}}{\partial z} + Q_{o} \left( {T_{o} - T_{\infty } } \right)e^{{\left( { - m_{1} \left( {\sqrt {\frac{\Omega }{{v_{f} \left( {1 - bt} \right)}}} z} \right)} \right)}} + \frac{{\mu_{hnf} }}{{(\rho c_{p} )_{hnf} }}\left[ {\left( {\frac{\partial u}{{\partial z}}} \right)^{2} - \left( {\frac{\partial v}{{\partial z}}} \right)^{2} } \right],$$

9 $$k^{**} (z) = k_{\infty } \left( {1 + d_{1} e^{{ - \frac{z}{\gamma }}} } \right),$$

10 $$\varepsilon (z) = \varepsilon_{\infty } \left( {1 + d_{2}^{*} e^{{ - \frac{z}{\gamma }}} } \right).$$

The radiative heat flux, which is given by:

11 $$q_{r} = - \frac{{4\sigma^{*} }}{{3k^{*} }}\frac{{\partial T^{4} }}{\partial z} = - \frac{{16T_{\infty }^{3} \sigma^{*} }}{{3k^{*} }}\left( {\frac{\partial T}{{\partial z}}} \right),$$

From Eqs. (11) and (8), one has

12 $$\begin{aligned} & \frac{\partial T}{{\partial t}} + u\frac{\partial T}{{\partial r}} + w\frac{\partial T}{{\partial z}} = \frac{{k_{hnf} }}{{(\rho c_{p} )_{hnf} }}\left( {\frac{{\partial^{2} T}}{{\partial z^{2} }}} \right) + \frac{{\mu_{hnf} }}{{(\rho c_{p} )_{hnf} }}\left[ {\left( {\frac{\partial u}{{\partial z}}} \right)^{2} - \left( {\frac{\partial v}{{\partial z}}} \right)^{2} } \right] \\ & \quad + \frac{{16\sigma^{*} T_{{_{\infty } }}^{3} }}{{3k^{*} (\rho c_{p} )_{hnf} }}\left( {\frac{{\partial^{2} T}}{{\partial z^{2} }}} \right) + Q_{o} \left( {T_{o} - T_{\infty } } \right)e^{{\left( { - m_{1} \left( {z\sqrt {\frac{\Omega }{{v_{f} \left( {1 - bt} \right)}}} } \right)} \right)}} . \\ \end{aligned}$$

where $$F = c_{b} /r\left( {k^{**} } \right)^{\frac{1}{2}}$$ represents non-uniform inertia factor, $$\varepsilon_{\infty }$$ stands for constant porosity, $$k_{\infty }$$ stands for constant permeability, $$d_{2}$$ for variable porosity, $$T$$ denotes fluid temperature, $$d_{1} \;$$ for the variable permeability, $$\sigma_{hnf}$$ is hybrid nanofluid electric conducting, $$k_{hnf}$$ denotes hybrid nanomaterials thermal conductivity, $$\rho_{hnf}$$ is the density, $$L_{1}$$ denotes velocity slip factor, $$Q_{o}$$ the heat generation/absorption parameter, $$c_{b}$$ is drag factor, $$k^{**}$$ is permeability of porous space, $$(\rho c_{p} )_{hnf}$$ is heat capacity of hybrid nanofluid, $$q_{r}$$ is radiative heat flux, $$m_{1}$$ the exponential index, $$\sigma^{*}$$ the coefficient of mean absorption, $$v_{hnf}$$ denotes kinematics viscosity, $$\gamma=\sqrt {\frac{v_{f} \left( {1 - bt} \right)}{\Omega }}$$ is the dimensional constant having dimension of length, and $$k^{*}$$ manifest the constant of Stefman Boltzmann.

The specified conditions are:

13 $$\begin{aligned} \left( {u,v,w,T} \right) & = \left( {L_{1} \frac{\partial u}{{\partial z}} + \frac{cr}{{\left( {1 - br} \right)}},\quad L_{1} \frac{\partial v}{{\partial z}} + \frac{r\Omega }{{\left( {1 - br} \right)}},\quad 0,\,T_{s} } \right)\quad at\,z\, = 0 \\ & \quad u = 0,v = 0,\quad T_{\infty } = T\quad as\,\,z \to \infty , \\ \end{aligned}$$

### Nano and Hybrid nanomaterials properties

The shape factor of nanoparticles is displayed in Table 1. The physical characteristics and relations of nano and hybrid nanomaterials are summarized in Tables 2 and 3 respectively.

**Table 1:** Shape factor of nanoparticles^50^.

Shape	Sphere	Tetrahedron	Cylinder	column	Lamina
Geometry					
Shape factor	3.0	4.6	4.9	6.3598	16.1676

**Table 2:** The nanoparticles $ZnO,\;CoF_{2} O_{4}$ and water thermo-physical features^51,52^.

	$\rho \,({\text{kg}}/{\text{m}}^{3} )$	$k\,({\text{W}}/{\text{mk}})$	$C_{p} \,({\text{J}}/{\text{kgK}})$	$\sigma \,(\Omega {\text{m}})^{ - 1}$
Pure water	997.1	0.613	4179	0.05
ZnO	5.606	19	544	0.01
CoF_2_O_4_	4907	3.7	700	5.51 $\times$ 10^9^

**Table 3:** Thermo-physical relations for nano and hybrid nanofluids^29,41^.

Properties	Nanofluid	Hybrid nanofluid
Density	$\rho_{nf} = \rho_{f} \left( {(1 - \phi_{1} ) + \phi_{1} \left( {\tfrac{{\rho_{s} }}{{\rho_{f} }}} \right)} \right)$	$$\begin{aligned} \rho_{hnf} & = \rho_{f} (1 - \phi_{1} )\left( {(1 - \phi_{2} ) + \phi_{1} \left( {\tfrac{{\rho_{ZnO} }}{{\rho_{f} }}} \right)} \right) \\ & \quad + \phi_{2} \rho_{CoF_2O_4} \\ \end{aligned}$$
Viscosity	$\mu_{nf} = \tfrac{{\mu_{f} }}{{(1 - \phi_{1} )^{2.5} }}$	$\mu_{nf} = \tfrac{{\mu_{f} }}{{(1 - \phi_{1} )^{2.5} (1 - \phi_{2} )^{2.5} }}$
Heat capacity	$(\rho c_{p} )_{nf} = (\rho c_{p} )_{f} \left( {1 - \phi_{1} + \phi_{1} \tfrac{{(\rho c_{p} )_{ZnO} }}{{(\rho c_{p} )_{f} }}} \right)$	$$\begin{aligned} & (\rho c_{p} )_{nf} = \phi_{2} \left( {\rho c_{p} } \right)_{CoF_2O_2} + (\rho c_{p} )_{f} (1 - \phi_{2} ) \\ & \left( {(1 - \phi_{1} ) + \phi_{1} \tfrac{{(\rho c_{p} )_{ZnO} }}{{(\rho c_{p} )_{f} }}} \right) \\ \end{aligned}$$
Thermal conductivity	$\frac{{k_{nf} }}{{k_{f} }} = \frac{{k_{ZnO} + (s - 1)k_{f} - (s - 1)\phi_{1} (k_{f} - k_{ZnO} )}}{{k_{ZnO} + (s - 1)k_{f} + \phi_{1} (k_{f} - k_{ZnO} )}}$	$$\begin{aligned} & \frac{{k_{hnf} }}{{k_{f} }} = \frac{{k_{ZnO} (s - 1)k_{f} - (s - 1)(k_{bf} - k_{CoF_2O_2} )\phi_{2} }}{{k_{ZnO} + (s - 1)k_{bf} + \phi_{2} (kb_{f} - k_{CoF_2O_2} )}} \\ & {\text{where}} \\ & \frac{{k_{bf} }}{{k_{f} }} = \frac{{k_{CoF_2O_2} + (s - 1)k_{f} - (s - 1)(k_{f} - k_{CoF_2O_2} )\phi_{1} }}{{k_{CoF_2O_2} + (s - 1)k_{f} + \phi_{1} (k_{f} - k_{CoF_2O_2} )}} \\ \end{aligned}$$
Electrical conductivity	$$\begin{aligned} & \frac{{\sigma_{nf} }}{{\sigma_{f} }} = 1 + \frac{{3\left( {\sigma - 1} \right)\phi_{1} }}{{\left( {\sigma + 2} \right) - \left( {\sigma - 1} \right)\phi_{1} }} \\ & {\text{where}}\,\sigma = \frac{{\sigma_{ZnO} }}{{\sigma_{f} }} \\ \end{aligned}$$	$$\begin{aligned} & \frac{{\sigma_{hnf} }}{{\sigma_{hf} }} = \frac{{\sigma_{CoF_2O_2} + 2\sigma_{bf} - 2\phi_{2} \left( {\sigma_{bf} - \sigma_{CoF_2O_2} } \right)}}{{\sigma_{CoF_2O_2} + 2\sigma_{bf} + \phi_{2} \left( {\sigma_{bf} - \sigma_{CoF_2O_2} } \right)}} \\ & {\text{where}}\,\frac{{\sigma_{bf} }}{{\sigma_{f} }} = \frac{{\sigma_{ZnO} + 2\sigma_{f} - 2\phi_{1} \left( {\sigma_{f} - \sigma_{ZnO} } \right)}}{{\sigma_{ZnO} + 2\sigma_{f} + \phi_{1} \left( {\sigma_{f} - \sigma_{ZnO} } \right)}} \\ \end{aligned}$$

### Transformations

The following transformations are introduce for current study^30^:

14 $$\begin{aligned} & \eta = \sqrt {\frac{\Omega }{{v_{f} \left( {1 - bt} \right)}}z} ,v = \frac{r\Omega }{{\left( {1 - bt} \right)}}g\left( \eta \right) \\ & u = \frac{r\Omega }{{\left( {1 - bt} \right)}}f^{\prime}\left( \eta \right),\quad T = T_{o} - T_{ref} \left( {\frac{{r^{2} \Omega }}{{v_{f} \left( {1 - bt} \right)^{\frac{3}{2}} }}} \right)\theta \left( \eta \right) \\ & w = - 2\left( {\frac{{v_{f} \Omega }}{1 - bt}} \right)^{\frac{1}{2}} f\left( \eta \right), \\ \end{aligned}$$

Employing the Eq. (11) in Eqs. (5–12) becomes

15 $$\begin{aligned} & \frac{{A_{1} }}{{A_{2} }}f^{\prime\prime\prime} + (g^{2} + 2ff^{\prime\prime} - f^{{\prime}{2}} ) - S\left( {\frac{\eta }{2}f^{\prime\prime} + f^{\prime}} \right) - \frac{{A_{3} }}{{A_{2} (1 + m^{2} )}}M(f^{\prime} - mg) \\ & \quad - \frac{{A_{1} }}{{A_{2} }}\frac{1}{{{\text{Re}}_{r} \alpha }}\left( {\frac{{1 + d_{2} e^{ - \eta } }}{{1 + d_{1} e^{ - \eta } }}} \right)f^{\prime} - F_{r} \frac{{\left( {1 + d_{2} e^{ - \eta } } \right)^{2} }}{{\sqrt {1 + d_{1} e^{ - \eta } } }}\left( {f^{{\prime}{2}} + g^{2} } \right) = 0, \\ \end{aligned}$$

16 $$\begin{aligned} & \frac{{A_{1} }}{{A_{2} }}g^{\prime\prime} - 2(fg^{\prime} - f^{\prime}g) - S\left( {\frac{\eta }{2}g^{\prime} + g} \right) - \frac{{A_{3} }}{{A_{2} (1 + m^{2} )}}M(g - mf^{\prime}) \\ & \quad - \frac{{A_{1} }}{{A_{2} }}\frac{1}{{{\text{Re}}_{r} \alpha }}\left( {\frac{{1 + d_{2} e^{ - \eta } }}{{1 + d_{1} e^{ - \eta } }}} \right)g - F_{r} \frac{{\left( {1 + d_{2} e^{ - \eta } } \right)^{2} }}{{\sqrt {1 + d_{1} e^{ - \eta } } }}\left( {f^{{\prime}{2}} + g^{2} } \right) = 0, \\ \end{aligned}$$

17 $$\begin{aligned} & A_{4} \left( {A_{5} + \frac{4}{3}Rd} \right)\theta^{\prime\prime} - \Pr S\left( {\frac{\eta }{2}\theta^{\prime} + \frac{3}{2}\theta } \right) + 2\Pr (f\theta^{\prime} - f^{\prime}\theta ) \\ & \quad + \Pr Q_{E} e^{{( - m_{1} \eta )}} - \frac{{A_{4} }}{{A_{1} }}\Pr Ec(f^{{\prime\prime}{2}} + g^{{\prime}{2}} ) = 0. \\ \end{aligned}$$

With

18 $$\begin{aligned} f & = 0,\quad f^{\prime} = \omega + \gamma_{s} f^{\prime\prime}(0),\quad g = 1 + \gamma_{s} g^{\prime}(0),\quad \theta \left( 0 \right) = 1,\quad at\,\,\eta = 0, \\ f^{\prime} & = 0,\quad g = 0,\quad \theta = 0,\quad as\,\,\eta \to 0, \\ \end{aligned}$$

where, $F_{r}$ stands for local inertia variable, $\alpha$ for porosity parameter, $\,\omega$ is rotational variable, $\,S$ measure unsteadiness, $M$ is magnetics variable, $m$ denotes Hall current variable, $\gamma_{s}$ is slip parameter, $\,Rd$ denotes radiation parameter, $\Pr$ is Prandtl number $\Pr = 6.5$ for water, $F_{r}$ denotes Forchheimer number and $Ec$ stands for Eckert number. The dimensionless variables and $A_{1} ,\,A_{2} ,\,A_{3} ,\,A_{4}$ and $A_{5}$ are expressed as:

19 $$  \begin{aligned}   Q_{{_{E} }}  &  = \frac{{Q_{0} }}{{\left( {\rho _{f} c_{p} } \right)_{{hnf}} \Omega }},\quad \gamma _{s}  = L_{1} \sqrt {\frac{\Omega }{{v_{f} \left( {1 - bt} \right)}}} ,\quad F_{r}  = \frac{{\varepsilon _{\infty } rc_{b} }}{{\sqrt {k_{\infty } } }},\quad \alpha  = \frac{{k_{\infty } }}{{r^{2} \varepsilon _{\infty } }},\quad S = \frac{b}{\Omega },\quad \omega  = \frac{\Omega }{c},\quad m = \omega _{e} \tau _{e} , \\    M &  = \frac{{\sigma _{1} B_{o}^{2} }}{{\rho _{f} \Omega }},\quad \Pr  = \frac{{\mu _{f} \left( {\rho C_{p} } \right)_{f} }}{{\rho _{f} k_{f} }},\quad Rd = \frac{{4\sigma ^{*} \left( {T_{{_{\infty } }}^{3} } \right)}}{{k^{*} k_{f} }},\quad Ec = \frac{{r^{2} \Omega ^{2} }}{{\left( {T_{s}  - T_{o} } \right)\left( {1 - bt} \right)^{2} }},\quad A_{1}  = \frac{{\mu _{{hnf}} }}{{\mu _{f} }}, \\    A_{2}  &  = \frac{{\rho _{{hnf}} }}{{\rho _{f} }},\quad A_{3}  = \frac{{\sigma _{{hnf}} }}{{\sigma _{f} }},\quad A_{4}  = \frac{{\left( {\rho C_{p} } \right)_{f} }}{{\left( {\rho C_{p} } \right)_{{hnf}} }},\quad A_{5}  = \frac{{k_{{hnf}} }}{{k_{f} }} \\  \end{aligned} $$

### Engineering quantities

The surface transport aspects focusing the current hybrid nanomaterials flow is inspected locally with help of skin frictions ($C_{fr}$, $C_{gr}$) and Nusselt number ($Nu_{r}$) as follows:

20 $$\begin{aligned} C_{fr} & = \frac{{2\mu_{hnf} \left( {\frac{\partial u}{{\partial z}}} \right)_{z = 0} }}{{\rho_{hnf} \left( {\frac{r\Omega }{{1 - bt}}} \right)^{2} }}, \\ C_{gr} & = \frac{{2\mu_{hnf} \left( {\frac{\partial v}{{\partial z}}} \right)_{z = 0} }}{{\rho_{hnf} \left( {\frac{r\Omega }{{1 - bt}}} \right)^{2} }}, \\ Nu_{r} & = \frac{{rq_{w} }}{{k_{hnf} (T - T_{\infty } )}}+(q_{r})_{z = 0}, \\ \end{aligned}$$

where $q_{w}$ designates the heat flux. After simplifications, the reduced quantities are:

21 $$\begin{aligned} {\text{Re}}^{\frac{1}{2}} Cf_{r} & = \frac{{f^{\prime\prime}\left( 0 \right)}}{{\left( {1 - \phi_{Ni} } \right)^{2.5} \left( {1 - \phi_{ZnO} } \right)^{2.5} }}, \\ {\text{Re}}^{\frac{1}{2}} Cg_{r} & = \frac{{g^{\prime}\left( 0 \right)}}{{\left( {1 - \phi_{Ni} } \right)^{2.5} \left( {1 - \phi_{ZnO} } \right)^{2.5} }}, \\ {\text{Re}}^{{ - \frac{1}{2}}} Nu_{r} & = - \left( {\frac{{k_{hnf} }}{{k_{f} }}} \right)\left( {1 - \frac{4}{3}Rd} \right)\theta^{\prime}\left( 0 \right). \\ \end{aligned}$$

where ${\text{Re}} = \frac{{r^{2} \Omega }}{{v_{f} \left( {1 - bt} \right)}}$ present the Reynold number.

## Methodology and validation of outcomes

The converted system in Eqs. (15–19) are non-linear and coupled, therefore close form solution is indeed difficult^9^. To side up this issue, the transform ODEs are treated numerically via NDSolve technique, adopting the software Mathematica. Basically NDSolve is a built-in shooting technique, which can be process for small step sizes leads to less error. Present fallouts with published outcomes are compared to validate the current problem (see Table 4). It is identified that an excellent match between present outcomes and Yin et al.^53^ and Turkyilmazoglu^54^ is obtained.

**Table 4:** Comparative studies of $- \theta^{\prime}(0)$,$- g^{\prime}(0)$ and $f^{\prime}(0)$ with^53,54^.

	Yin et al.^53^	Turkyilmazoglu^54^	Present outcomes
$f^{\prime}(0)$	0.51022941	0.51023262	0.51024311
$- g^{\prime}(0)$	0.61591990	0.61592201	0.61591032
$- \theta^{\prime}(0)$	0.93387285	0.93387794	0.93376327

## Physical discussion of outcomes

**Figure 2:**
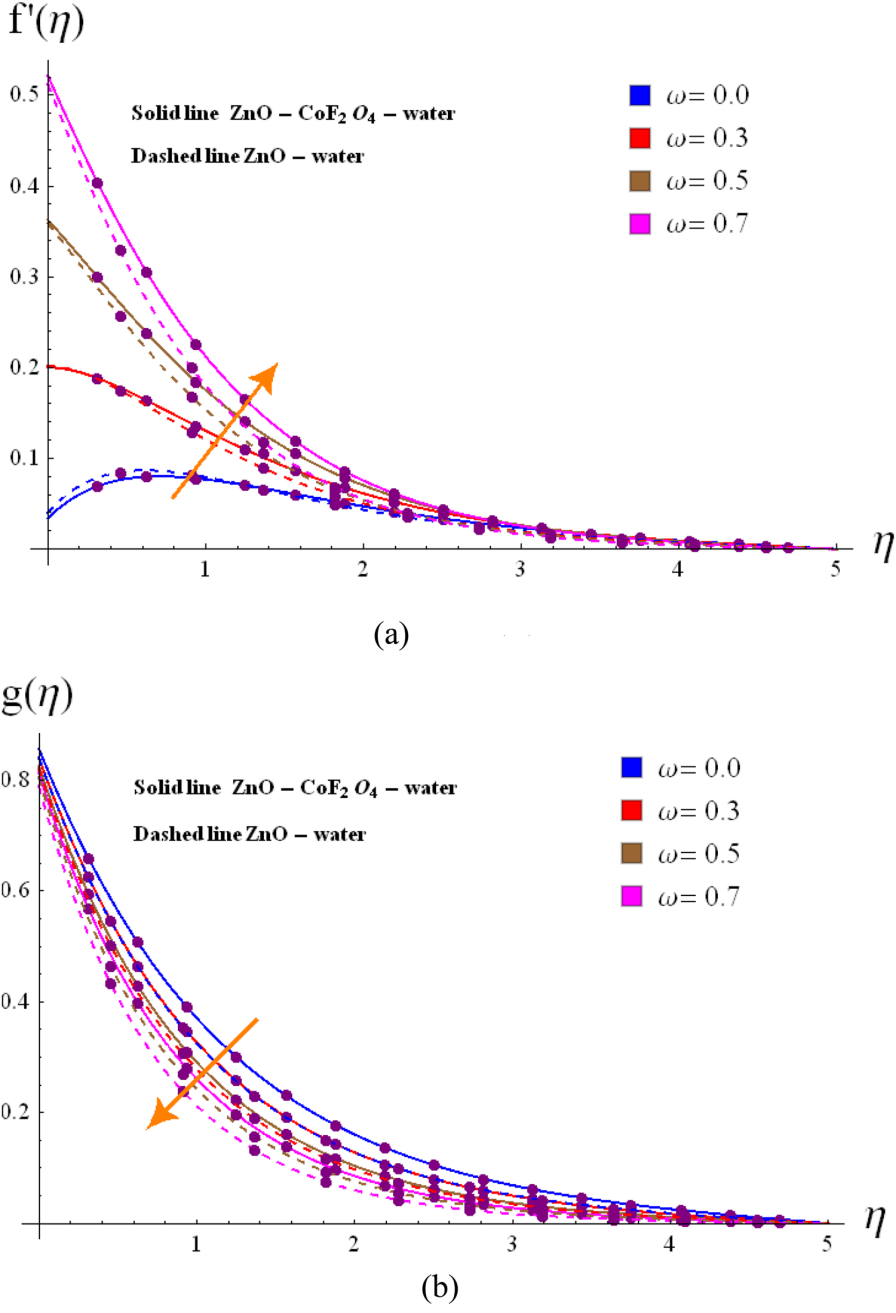
(**a**) Variation of $f^{\prime}\left( \eta \right)$ via $\omega$. (**b**) Variation of $g\left( \eta \right)$ via $\omega$.

**Figure 3:**
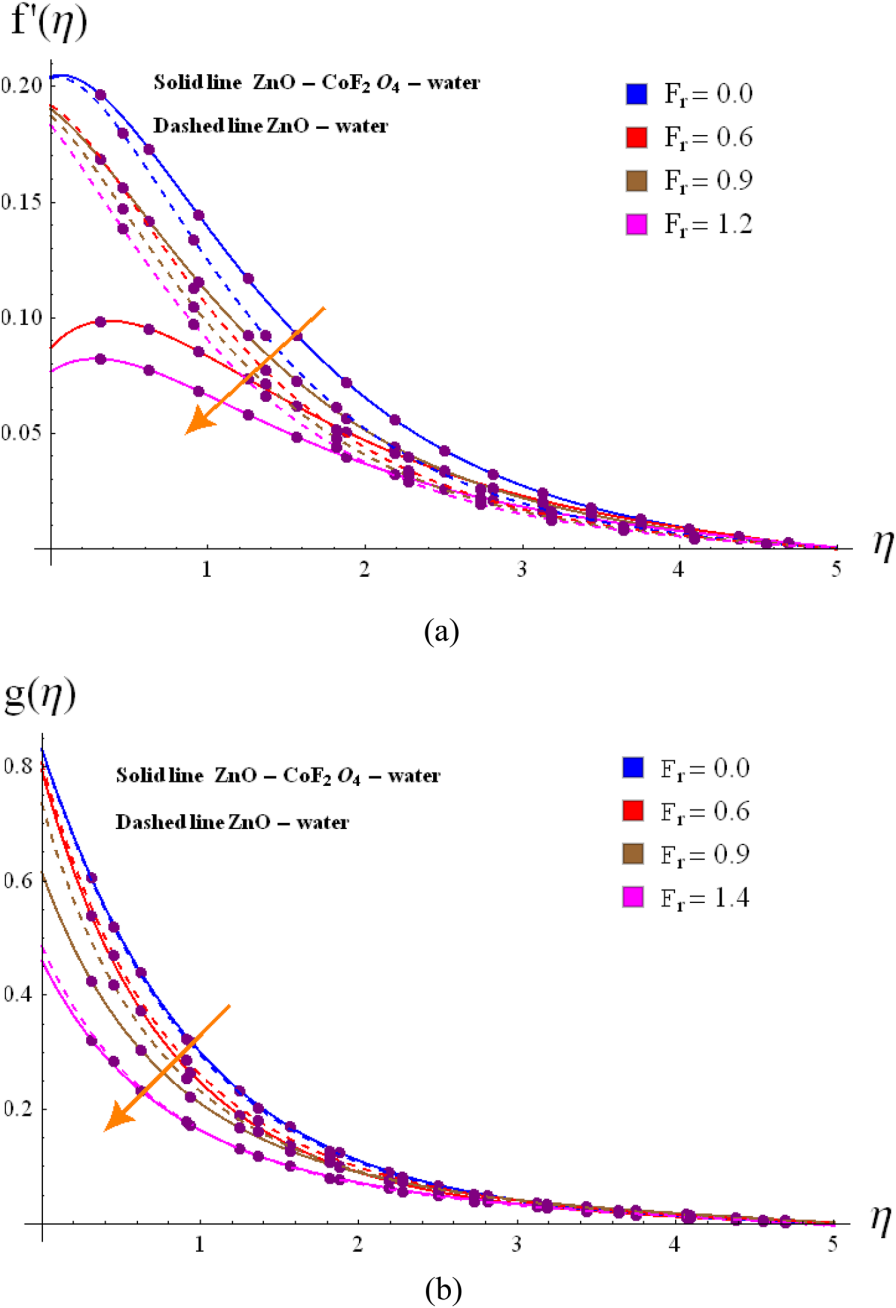
(**a**) Variation of $f^{\prime}\left( \eta \right)$ via $F_{r}$. (**b**) Variation of $g\left( \eta \right)$ via $F_r$.

**Figure 4:**
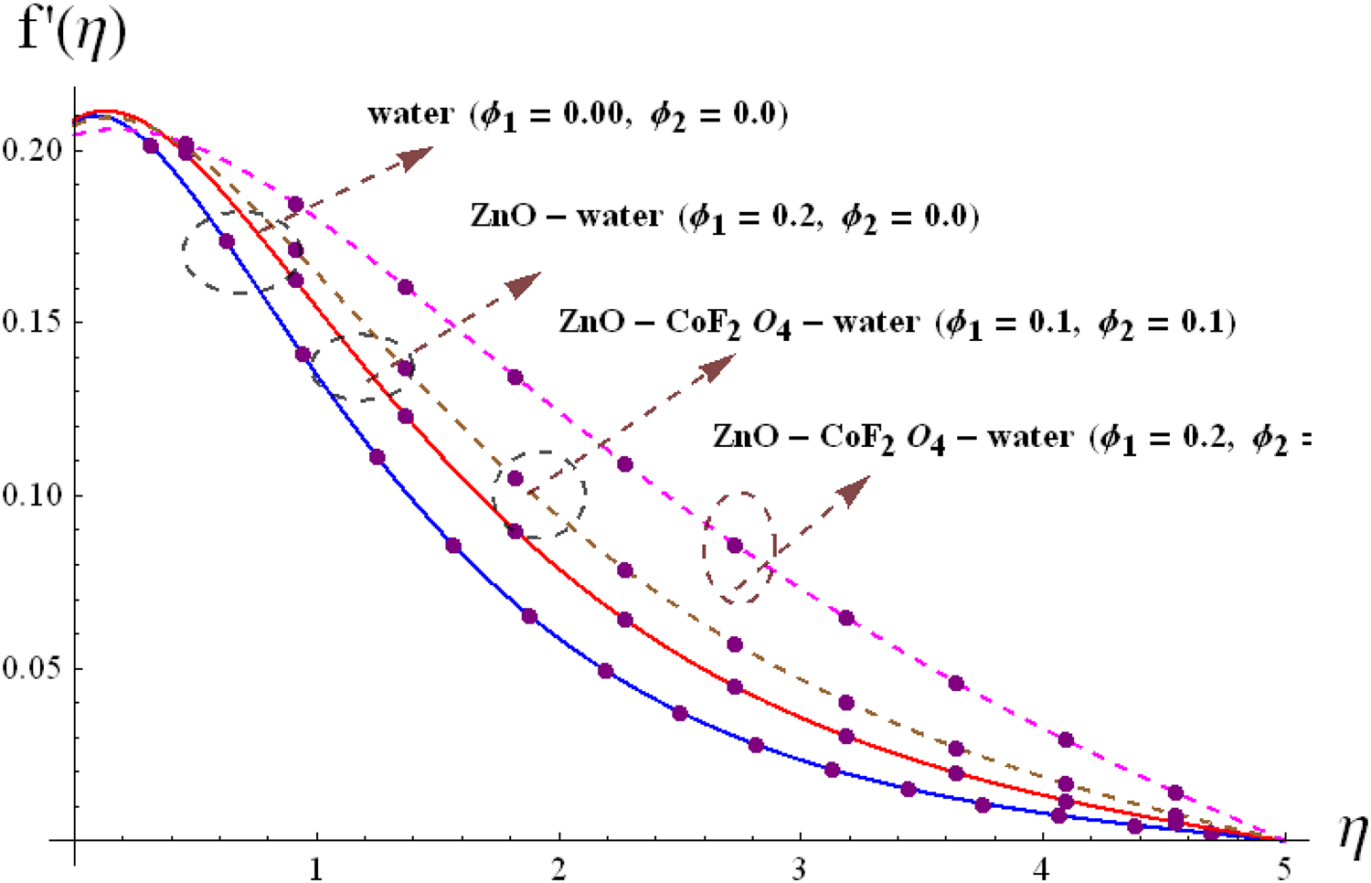
Variation of $f^{\prime}\left( \eta \right)$ via $\phi_{1} /\phi_{2}$.

**Figure 5:**
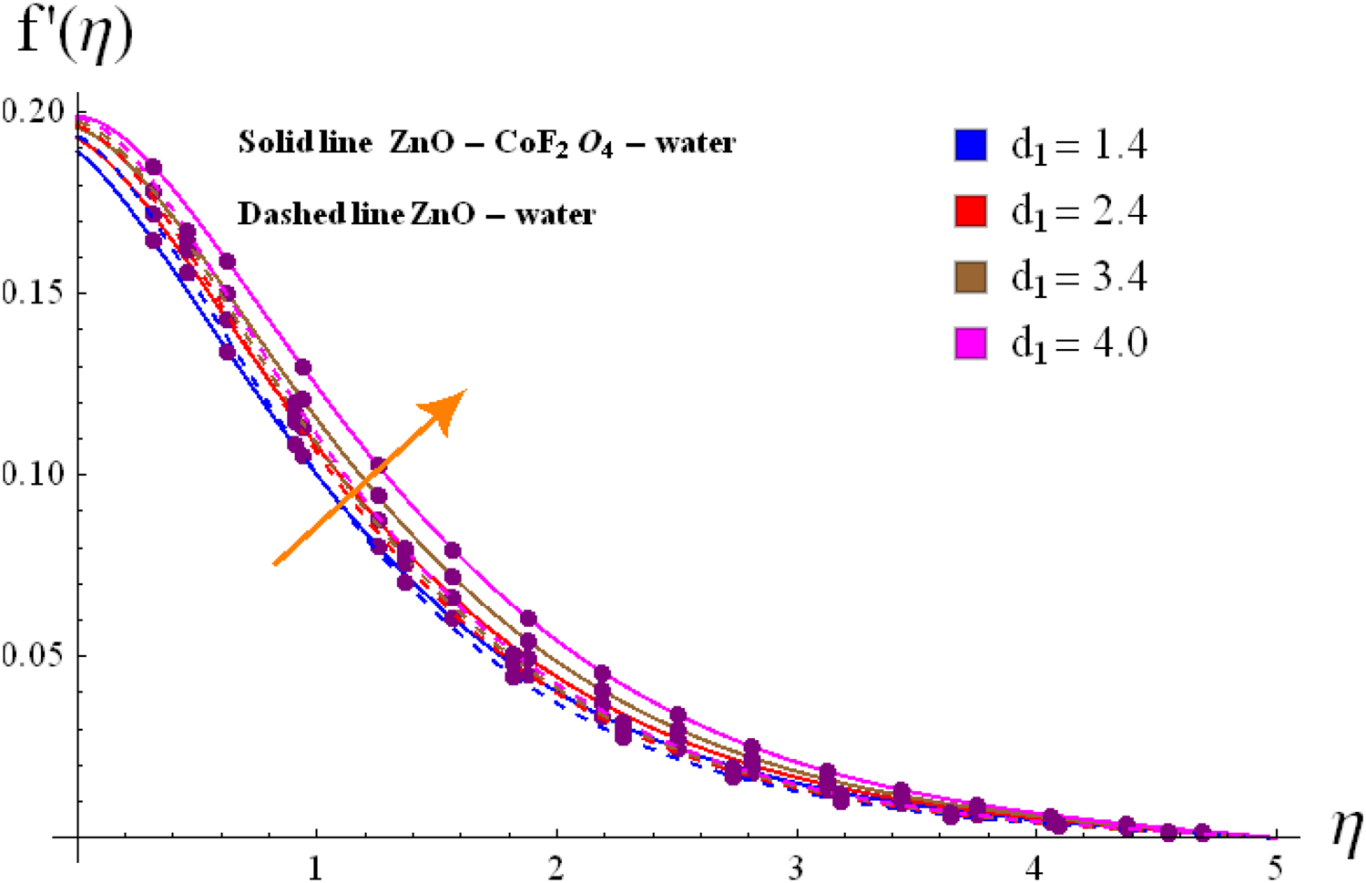
Variation of $f^{\prime}\left( \eta \right)$ via $d_1$.

**Figure 6:**
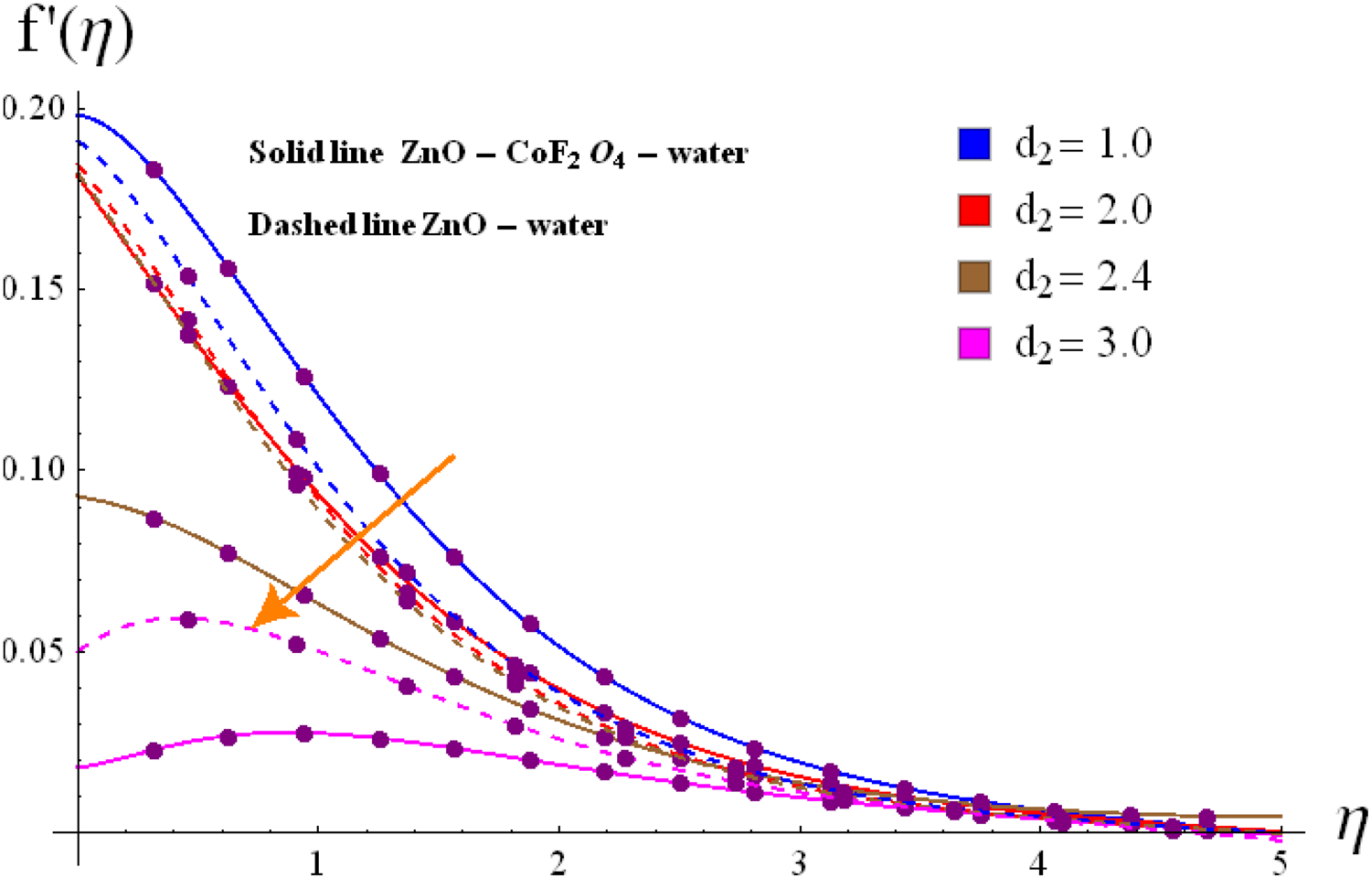
Variation of $f^{\prime}\left( \eta \right)$ via $d_2$.

**Figure 7:**
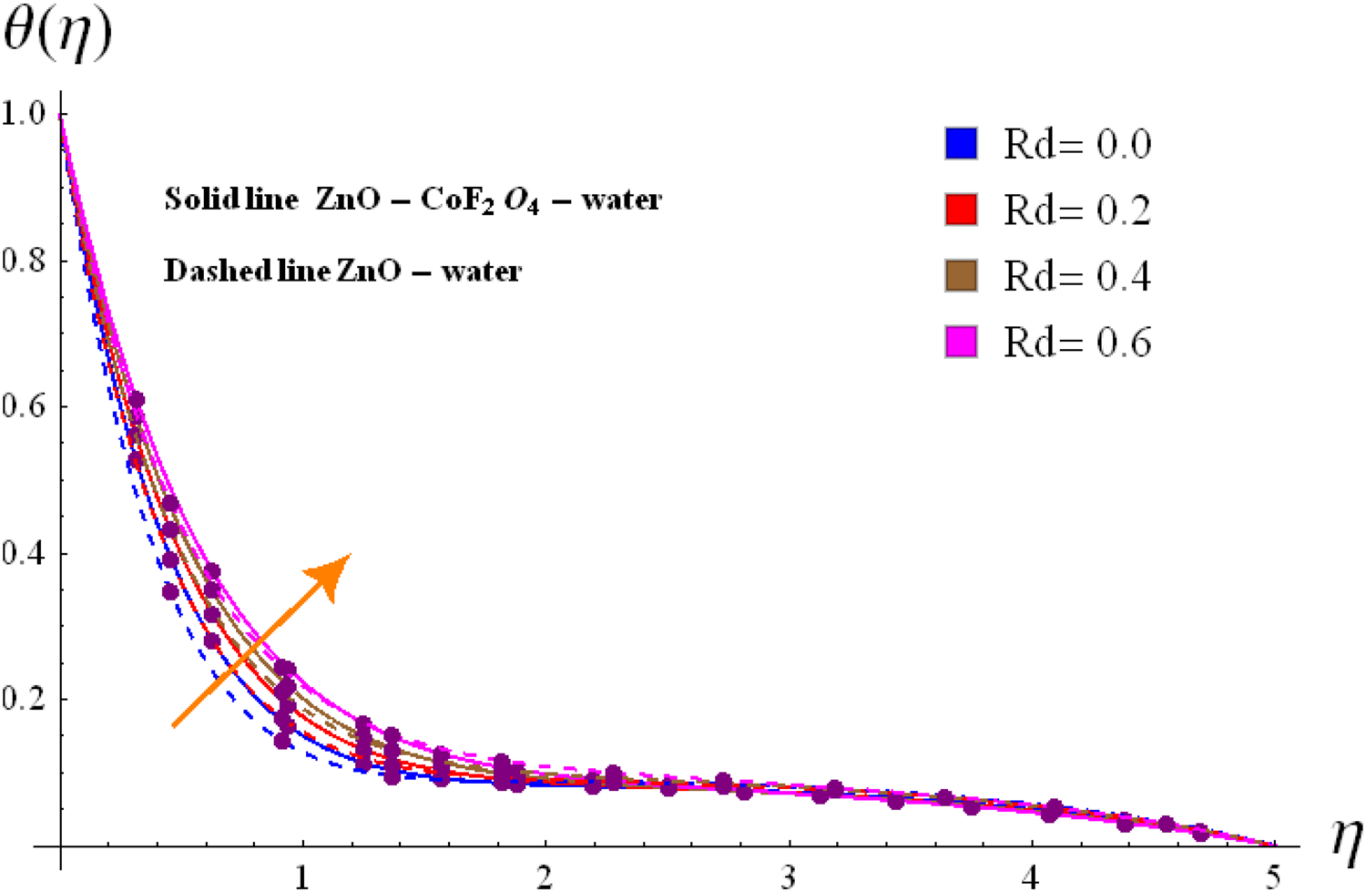
Variation of $\theta \left( \eta \right)$ via $Rd$.

**Figure 8:**
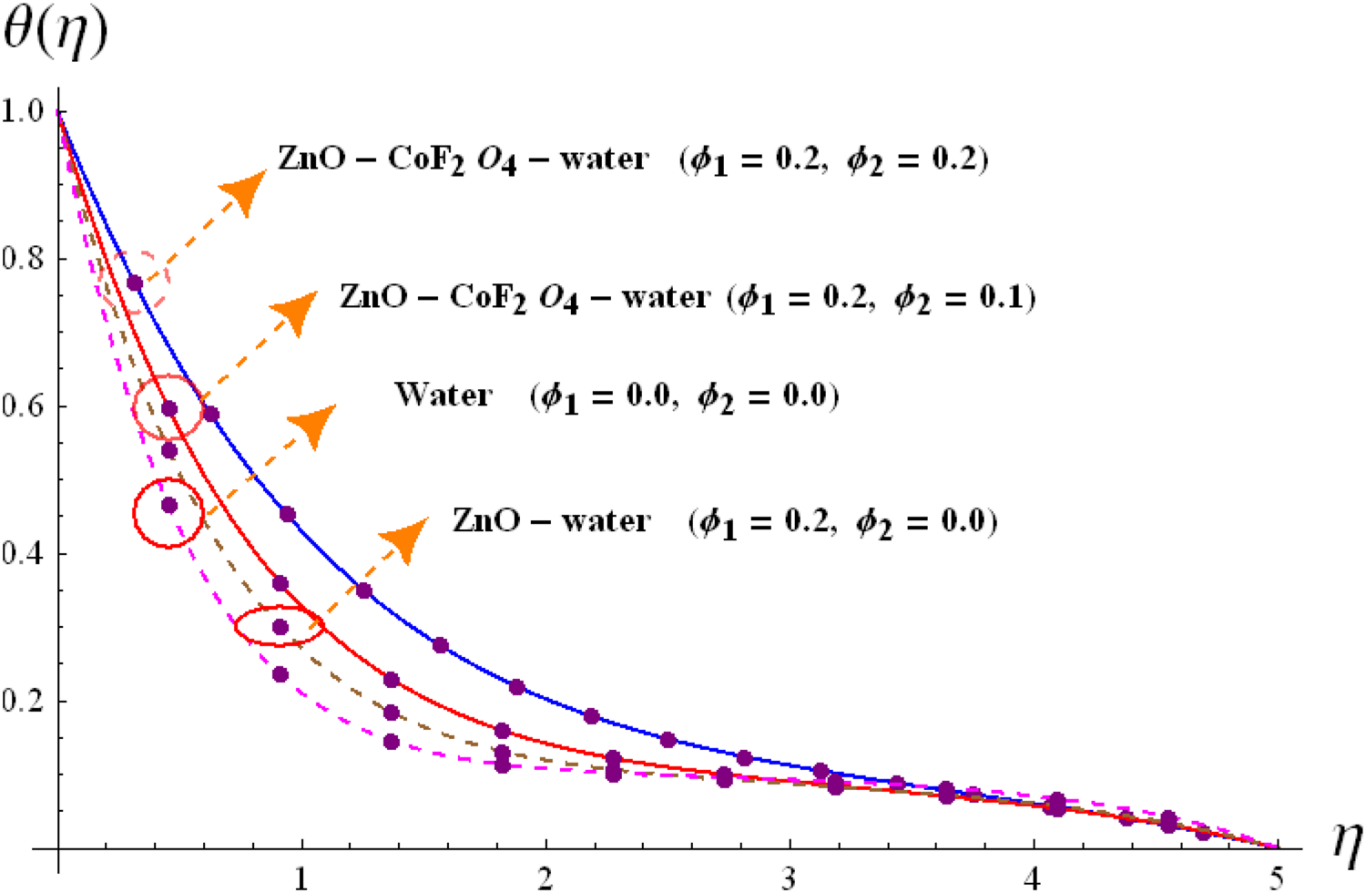
Variation of $\theta \left( \eta \right)$ via $\phi_{1} /\phi_{2}$.

Base on the previous numerical executions, many illustrative outcomes are designed in this section as depicted in Figs. 2, 3, 4, 5, 6, 7 and 8 for $f^{\prime}\left( \eta \right)$,$g\left( \eta \right)$, $\theta \left( \eta \right)$ and $C_{f,r}$ and $Nu_{r}$ against various estimations of interesting variables. For whole study, the default values of physical parameters are $\omega = 0.2$, $\gamma_{s} = 0.2$, $M = 0.2$, $F_{r} = 0.1$, $\alpha = 0.2, d_1=d_2=0.5$, $m = 0.2$, $Rd = 0.2$, $m_{1} = 0.1$, $Q_{E} = 0.25$, $Ec = 0.2$, $\phi_{1} = 0.01$, and $\phi_{2} = 0.01$. Some numerical values are assigning to each parameter, while other parameters are kept unchanged. Curves for hybrid and nano phases are denoted by solid and dished lines respectively.

### Velocity interpretation

The radial $f^{\prime}\left( \eta \right)$ and tangential $g\left( \eta \right)$ velocities against $\omega$, $Fr$, $d_{1}$ $d_{2}$ and $\phi_{1} /\phi_{2}$ are shown in Figs. 2, 3, 4, 5, 6, 7 and 8. Results for radial velocity $f^{\prime}\left( \eta \right)$ versus $\omega$ is presented in Fig. 2a. Here rising the estimations of $\omega$ leads to enhance the nanofluid velocity. Rotation variable is the ratio of rotating rate to stretching rate. Thus, larger estimations of $\omega$ implies higher rotation rate when compared with rate of stretching. In fact an increment in $\omega$ means enhancing the centrifugal force which consequently deploy pressure on nanomaterials to boosts up the motion of liquid particles in the radiation direction, where the decaying behavior for tangential velocity $g\left( \eta \right)$ is seen in Fig, 2b. Fig, 3a, b illustrates the impact of $Fr$ on $f^{\prime}\left( \eta \right)$ and $g\left( \eta \right)$. It is noted that higher $Fr$ corresponds decline $f^{\prime}\left( \eta \right)$ and $g\left( \eta \right)$ in both nano and hybrid phases. In fact higher $Fr$ leads to higher inertial force which decays both velocities. Comparative analysis of hybrid $\left( {\phi_{1} \not = 0,\,\,\phi_{2} \not = 0} \right)$ and base liquid $\left( {\phi_{1} = 0,\,\,\phi_{2} = 0} \right)$ on radial velocity $f^{\prime}\left( \eta \right)$ is illustrtared in in Fig. 4. Clearly hybrid nanofluid $\left( {\phi_{1} \not = 0,\,\,\phi_{2} = 0} \right)$ have more parts in rising $f^{\prime}\left( \eta \right)$ than the nano and base liquids. Figure 5 is schemed graphically to explore the dynamical features of hybrid and nano phases against $d_{1}$. Boosting trend is observed in this sketch with growing estimation of $d_{1}$.

Contrarily to the aforementioned impact, radial velocity $f^{\prime}\left( \eta \right)$ shows diminishing behavior against $d_{2}$ for both cases of nanofluids.

### Temperature interpretation

Figures 7 and 8 are designed to investigate the comparative analysis nano and hybrid nanomaterials on $\theta \left( \eta \right)$ against varying estimations of $Rd$ and $\phi_{1} /\phi_{2}$**.** The variation of $Rd$ on $\theta \left( \eta \right)$ for hybrid and nano particles are structured in Fig. 7. It is shown that sharply increment in $Rd$ boosts up thermal field due to more heat absorbed by hybrid nanofluid. Physically, higher $Rd$ means more heat is provided to hybrid nanomaterials, that is why $\theta \left( \eta \right)$ enhances. The variation of $\theta \left( \eta \right)$ of hybrid nanoliquid for rising estimations of $\phi_{1} /\phi_{2}$ is shaped in Fig. 8. Higher $\phi_{1} /\phi_{2}$ leads to boost the nano and hybrid nanoliquid thermal field. This figure also provide us comparative study for three different cases i.e. hybrid $\left( {\phi_{1} = 0,\,\,\phi_{2} \not = 0} \right)$, nano $\left( {\phi_{1} \not = 0,\,\,\phi_{2} = 0} \right)$ and base $\left( {\phi_{1} = 0,\,\,\phi_{2} = 0} \right)$. Clearly hybrid nanoliquid dominates over the base and nano liquids.

### Variation in physical quantities

The skin frictions of the hybrid and nano phases through distinct variables are disclosed in Table 5. It is detected that $Cf_{r}$ and $Cg_{r}$ have opposite trend for $d_{1}$ and $d_{2}$. Furthermore, skin frictions are more in case of hybrid nanoliquid than the traditional nanoliquid. Table 6 designed the variations in Nusselt number with shape factor against several variables. Here it is seen that heat transfer rate is higher for lamina shaped nanoparticles.

**Table 5:** Skin frictions ($Cf_{r}$ and $Cg_{r}$) numerical values versus various parameter.

$d_{1}$	$d_{2}$	$\lambda$	$F_{r}$	$\omega$	$Cf_{r}$		$Cg_{r}$	
					$ZnO\,Water$	$ZnO - CoF_{2} O_{4} \,Water$	$ZnO\,Water$	$ZnO - CoF_{2} O_{4} \,Water$
0.2	2.0	0.2	0.2	0.2	0.039018	0.055687	0.969356	1.138655
0.5					0.031256	0.046169	0.959022	1.123875
2.0					0.010282	0.011103	0.933633	1.075667
5.2	0.0	0.2	0.2	0.2	0.012797	0.007865	0.911227	1.055545
	0.2				0.005854	0.000062	0.916651	1.062424
	0.4				− 0.00185	-0.00853	0.922603	1.066083
5.2	0.2	0.0	0.2	0.2	0.040515	0.035413	0.901291	1.044377
		0.3			− 0.00957	− 0.03073	0.916651	1.062424
		0.6			− 0.000415	− 0.05778	0.9314307	0.984552
5.2	0.2	0.2	0.0	0.2	0.0314713	0.028414	0.8650194	1.004653
			0.2		0.0058540	0.000062	0.9416488	1.117536
			0.4		− 0.018760	0.111325	0.1119701	1.169940
5.2	0.2	0.2	0.2	0.0	0.2042584	0.226319	0.8181331	0.953427
				0.3	− 0.101338	− 0.12255	0.9609267	1.111851
				0.6	− 0.451223	− 0.52400	1.0782713	0.524002

**Table 6:** Numerical data of (c$Nu_{r}$) versus various parameters and shape factor.

Parameters			$Nu_{r}$				
$Rd$	$d_{1}$	$Q_{E}$	Shape factors effect on hybrid nanofluid				
0.2	5.0	0.2	$s = 3$	$s = 4.6$	$s = 4.9$	$s = 6.3598$	$s = 16.1576$
0.0			2.669020	2.833824	2.86322	3.000252	3.72986
0.2			3.067484	3.292329	3.33232	3.518228	4.49681
0.4			3.418238	3.698038	3.74787	3.979717	5.20179
0.2	0.0	0.2	2.450075	1.274921	2.35187	3.414902	4.34628
	0.2		2.994185	1.186629	2.27522	3.427278	4.36405
	0.5		3.006047	1.110452	3.26226	3.441933	4.38519
0.2	5.0	0.0	0.456489	0.664149	0.77614	1.145939	4.84184
		0.1	3.067484	3.292329	3.33232	3.518264	4.49682
		0.2	2.834797	3.041641	3.07843	3.249546	4.15181

## Concluding remarks

Here significant features of variable porosity and permeability on hybrid nanofluid $$CoF_{2} O_{4} - ZnO/water)$$ flow through Darcy–Forchheimer space with the impact of radiation, EHS and hall current is addressed. Main findings of current analysis are:

- Variable porosity and permeability have reverse behavior on $f^{\prime}\left( \eta \right)$.
- Both radial and tangential velocities have opposite behavior for higher $\omega$.
- Temperature is increasing for $\phi_{1} /\phi_{2}$ and $Rd$.
- Thermal field is higher for ($$CoF_{2} O_{4} - ZnO/water)$$ than ($$ZnO/H_{2} O$$) nanomaterials.
- Nusselt number is grows up more rapidly in case of lamina shape nanoparticles in comparison with other shape nano particles.
- Hybrid nanomaterials have dominant effect thought out the analysis than the ordinary one.
- Lamina shape of nanoparticle is more effective and improves the thermal field than other shapes.
- Present analysis can be extended by incorporating entropy analysis, ternary hybrid nanofluid, fractional modeling and different techniques as future work^55–66^.

## List of symbols

$u,\;v,w$: Components of velocity
$$\Omega$$: Angular velocity
${\text{Re}}_{r}$: Local Reynolds number
$\sigma^{*}$: Coefficient of mean absorption.
$k^{*}$: Steffman Biltzmann constant
$$L_{1}$$: Slip factor
$$\Pr$$: Prandtl number
$\rho$: Density of fluid
$$CoF_{2} O_{4}$$: Cobalt ferrite
$g\left( \eta \right)$: Dimensionless axial velocity
$m$: Hall current parameter
$Q_{E}$: Heat source variable
$q_{r}$: Radiative heat flux
$$p_{e}$$: Electron pressure
$$m_{1}$$: Exponential index
$$\nu$$: Kinematic viscosity
$$nf$$: Nano fluid
EHS: Exponential heat source
$$\omega$$: Rotational variable
$B_{o}$: Magnetic field strength
$$hnf$$: Hybrid nanofluid
$$f$$: Fluid
$\mu_{e}$: Electron magnetic permeability
$$T_{o}$$: Origin temperature
$s$: Shape factor of nanoparticles
$C_{fr}$: Skin friction in radial direction
$$r,\phi ,z$$: Cylindrical coordinates
$\sigma$: Fluid electrical conductivity
$$T_{s}$$: Surface temperature
$$k$$: Thermal conductivity
$$Ec$$: Ecket number
$$c_{b}$$: Drag factor
$$Nu_{r}$$: Nusselt number
$\tau_{e}$: Electron collision time
$\theta \left( \eta \right)$: Dimension less temperature
$F_{r}$: Local inertia parameter
$f^{\prime}(\eta )$: Dimensionless radial velocity
$$ZnO$$: Zinc oxide
$$k^{**}$$: Permeability of porous space
$$\infty$$: Ambient condition
$$d_{1} \;$$: Variable permeability
$$c_{p}$$: Specific heat
c: Stretching rate
$b$: Dimensional positive constant
$$\varepsilon_{\infty }$$: Constant porosity
$$d_{2}$$: Variable porosity
$$k_{\infty }$$: Constant permeability
$\omega_{e}$: Cyclotron frequency of electron
$$T$$: Fluid temperature
$Q_{0}$: Heat generation/absorption
$$\gamma_{s}$$: Slip parameter
$C_{gr}$: Skin friction in tangential direction
